# Consequences of a high phosphorus intake on mineral metabolism and bone remodeling in dependence of calcium intake in healthy subjects – a randomized placebo-controlled human intervention study

**DOI:** 10.1186/s12937-016-0125-5

**Published:** 2016-01-19

**Authors:** Ulrike Trautvetter, Gerhard Jahreis, Michael Kiehntopf, Michael Glei

**Affiliations:** 1 Department of Nutritional Toxicology, Institute of Nutrition, Friedrich Schiller University Jena, Dornburger Straße 24, 07743 Jena, Germany; 2 Department of Nutritional Physiology, Institute of Nutrition, Friedrich Schiller University Jena, Dornburger Straße 24, 07743 Jena, Germany; 3 Institute of Clinical Chemistry and Laboratory Medicine, Jena University Hospitel, Erlanger Allee 101, 07747 Jena, Germany

**Keywords:** FGF23, Phosphorus intake, Calcium intake, Mineral metabolism, Human study

## Abstract

**Background:**

Epidemiological studies reported an association between plasma phosphate concentrations and a higher risk for death and cardiovascular events in subjects free of chronic kidney diseases. The main aims of the present study were to determine the influence of a high phosphorus intake in combination with different calcium supplies on phosphorus, calcium, magnesium and iron metabolism as well as fibroblast growth factor 23 (FGF23) concentrations within eight weeks of supplementation.

**Methods:**

Sixty-two healthy subjects completed the double-blind, placebo-controlled parallel designed study. Supplements were monosodium phosphate and calcium carbonate. During the first two weeks, all groups consumed a placebo sherbet powder, and afterwards, for eight weeks, a sherbet powder according to the intervention group: P1000/Ca0 (1 g/d phosphorus), P1000/Ca500 (1 g/d phosphorus and 0.5 g/d calcium) and P1000/Ca1000 (1 g/d phosphorus and 1 g/d calcium). Dietary records, fasting blood samplings, urine and fecal collections took place.

**Results:**

Fasting plasma phosphate concentrations did not change after any intervention. After all interventions, renal excretions and fecal concentrations of phosphorus increased significantly after eight weeks. Renal calcium and magnesium excretion decreased significantly after eight weeks of P1000/Ca0 intervention compared to placebo. Plasma FGF23 concentrations were significantly higher after four weeks compared to eight weeks of all interventions.

**Conclusions:**

The long-term study showed in healthy adults no influence of high phosphorus intakes on fasting plasma phosphate concentrations. A high phosphorus intake without adequate calcium intake seems to have negative impact on calcium metabolism. Plasma FGF23 concentrations increased four weeks after high phosphorus intake and normalized after eight weeks.

**Trial registration:**

The trial is registered at ClinicalTrials.gov as NCT02095392.

## Background

The association between elevated plasma phosphate concentrations and cardiovascular morbidity and mortality is well known in patients with chronic kidney disease (CKD) [1]. Nevertheless, in the last years, this association has been shown also for subjects without CKD and phosphate concentrations in the normal range. The Cholesterol And Recurrent Events (CARE) study as well as the Framingham Offspring Study showed a relation between serum phosphate concentrations >1.13 mmol/l and a higher risk for death and cardiovascular events in individuals free of CKD [2, 3]. However, the CARE and the Framingham Offspring Study have not determined the influence of dietary intake on serum phosphate.

In association with the phosphate metabolism in humans, the fibroblast growth factor 23 (FGF23) got more and more attention. Jüppner *et* Bergwitz [4] deemed FGF23 as a biomarker for the dysregulation of the phosphate balance in patients with CKD; this biomarker is probably involved in the pathophysiology of cardiovascular events in these patients. Together with vitamin D and parathyroid hormone (PTH), FGF23 equilibrates high plasma phosphate concentrations (after dietary intake) by increasing renal phosphate excretion and decreasing absorption of phosphate in the gut [5]. In the early stage of CKD, first FGF23 and then PTH increases to normalize serum phosphate concentration. But this mechanistic FGF23 increase is not yet fully understood [6]. Furthermore, the physiology of FGF23 in healthy adults after phosphate load is not sufficient examined.

Ritz et al. [7], Uribarri and Calvo [8] assumed, that the increased use of phosphorus (P) in food processing and the consequentially increased intake of P, which exceeds the nutritional recommendation, is responsible for the association with cardiovascular morbidity and mortality, even with serum phosphate concentrations in the normal range. Indeed, the recommendation of the US Institute of Medicine for adults between 19 and 50 years are 700 mg P/d, but the intake of P is far above this recommendation in the USA (approximately 1600 mg/d for men and 1200 mg/d for women) [9]. Due to the latest report of the European Food Safety Authority in Europe the phosphorus intakes ranges between 1000–1767 mg/d and are far above the recommendations, too [10]. Therefore, investigations of the consequences after high P intake in healthy humans is an important area of research.

Studies regarding high P intakes have some limitations, e.g., comparison of restricted versus supplemented P diet [11], short-term supplementation [12] and small sample size [13]. The present double-blind, placebo-controlled human intervention study in parallel design tries to compensate these former limitations and to complement the previous findings to get more insight and information about the consequences of a high P intake in healthy adults taking different calcium (Ca) intakes into consideration. The objectives of the present study are to determine the influence of high P intakes on phosphorus, calcium, magnesium and iron metabolism as well as FGF23 concentrations and bone remodeling marker.

## Methods

### Supplements

In the current study, two supplements were used: monosodium phosphate (NaH_2_PO_4_; cfb, Budenheim, Germany) and calcium carbonate (CaCO_3_; cfb, Budenheim, Germany). In order to achieve a supplementation of additional 1000 mg P/d as well as additional 500 or 1000 mg Ca/d, monosodium phosphate and calcium carbonate were added to sherbet powder. Sherbet powder without additional supplements served as the placebo. Taste and visual properties of placebo and test products were roughly comparable. Participants were encouraged to consume the sherbet powder twice a day diluted in 250 ml water. It contains: L(+)-tartaric acid, sodium hydrogen carbonate, sodium cyclamate, acesulfame-K, sodium saccharin, flavors and dyes. The dosage of two sherbet powders per day consisted of 138 kJ and 6.7 g carbohydrates.

### Subjects and study design

The study was conducted at the Friedrich Schiller University Jena, Department of Nutritional Toxicology, between March and July 2014.

Sixty-six omnivorous healthy subjects (men, *n* = 33; women *n* = 33) participated in this double-blind, placebo-controlled parallel designed study. Eligibility criteria for participants were age between 18 and 60 years and physical health. Exclusion criteria were regular intake of dietary supplements, renal diseases, pregnancy, nursing as well as post-menopausal age. Renal diseases were determined with the Chronic Kidney Disease Epidemiology Collaboration equation for estimating the glomerular filtration rate (CKD-EPI). Subjects with baseline glomerular filtration rates <80 ml/min/173m^2^ were excluded.

**Fig. 1:**
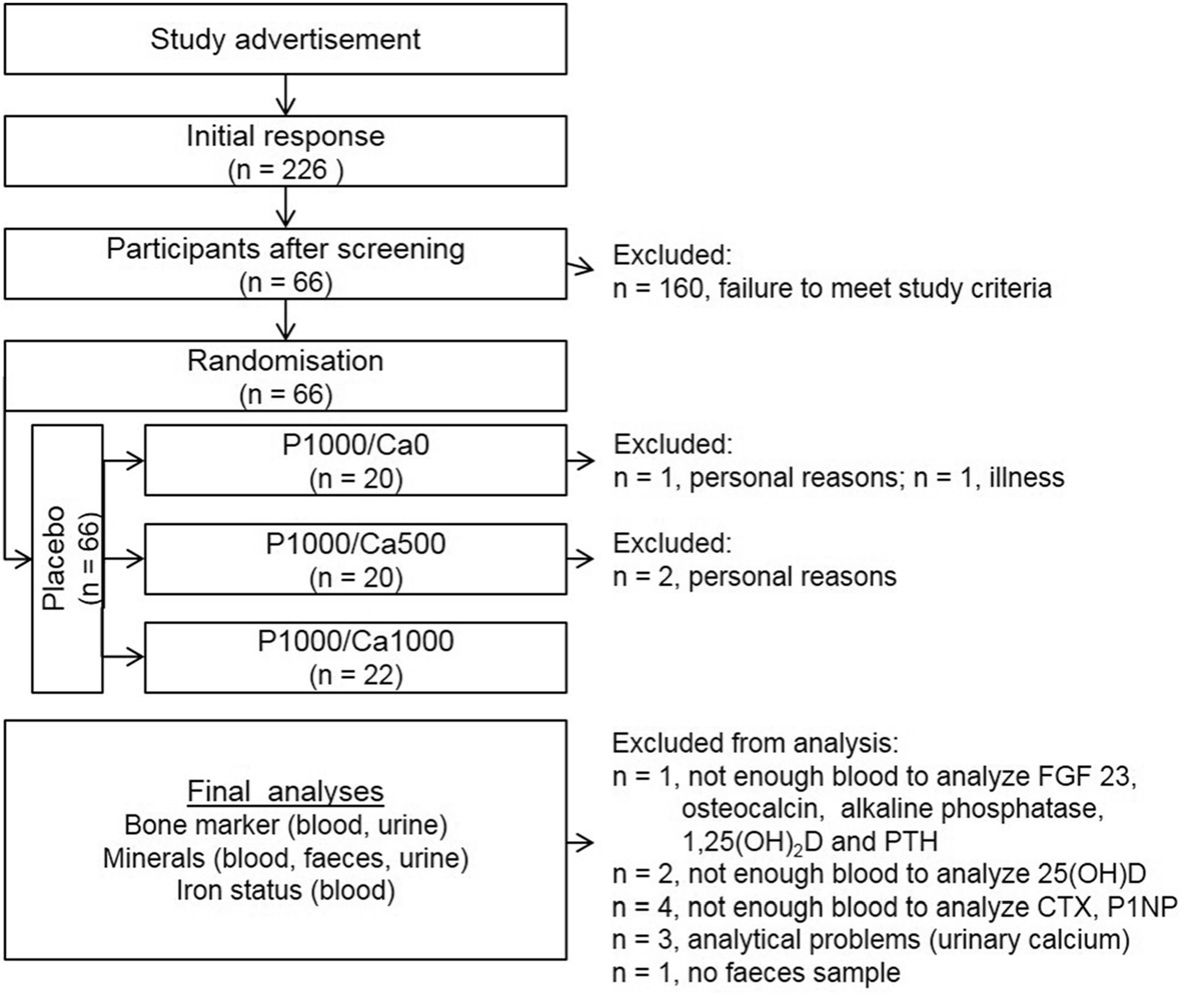
Flow chart of the study course. CTX: cross-linked C-terminal telopeptide of type I collagen; FGF23: fibroblast growth factor 23; PTH: parathyroid hormone; P1NP: N-terminal propeptide of type I procollagen; P1000/Ca0: 1000 mg phosphorus/0 mg calcium; P1000/Ca500: 1000 mg phosphorus/500 mg calcium; P1000/Ca1000: 1000 mg phosphorus/1000 mg calcium

The volunteers were provided with detailed information regarding purpose, course, and possible risks of the study. This study was conducted according to the guidelines laid down in the Declaration of Helsinki and all procedures involving human subjects were approved by the Ethical Committee of the Friedrich Schiller University Jena (No.: 3987-01/14 ). Written informed consent was obtained from all subjects. The trial is registered at ClinicalTrials.gov as NCT02095392. Four participants dropped out because of illness and personal reasons (Fig. 1). The remaining 62 volunteers (men, *n* = 30; women, *n* = 32) aged 29 ± 7 years and had a BMI of 24 ± 3 kg/m^2^.

**Fig. 2:**
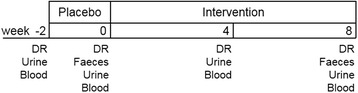
Timeline and content of sample collection. DR: dietary record; Intervention: 1000 mg phosphorus/0 mg calcium, 1000 mg phosphorus/500 mg calcium or 1000 mg phosphorus/1000 mg calcium

At the beginning of the study (baseline), all subjects documented their normal nutritional habits in a dietary record for seven successive days. The participants were encouraged to weigh all eaten foods with proved scales. Besides, a 24 h-urine collection and a fasting blood sampling took place. This procedure aimed to familiarize the subjects to the sample collection and to establish a baseline profile of each subject. After baseline, subjects were blinded allocated to three groups, so that there were no significant changes in age, BMI and 25-hydroxycholecalciferol (25(OH)D) concentration between the groups. Then groups were randomly assigned to the three intervention groups: P1000/Ca0, P1000/Ca500 or P1000/Ca1000 using simple randomization procedures (drawing lots). In the first two weeks, all subjects consumed the placebo product. Afterwards subjects consumed for eight weeks the respective supplement. In the last week of the placebo and after four and eight weeks of intervention, subjects document their nutritional habits in a dietary record for three days (two successive weekdays and one weekend day) and a 24 h-urine as well as a fasting blood sampling took place, too. Furthermore, subjects collected a feces sample after placebo and eight weeks of intervention (Fig. 2).

### Sample preparation

Participants and samples from each participant were coded to protect volunteer identity and to mask treatment groups during sample collection and analysis.

Blood samples were drawn by venipuncture and collected in lithium heparin, potassium EDTA and serum tubes. Potassium EDTA tubes were centrifuged (2500 x *g*, 10 min, 20 °C) and the plasma supernatants were stored at -80 °C until analysis. Serum and lithium heparin tubes were transported to the Institute of Clinical Chemistry and Laboratory Medicine, Jena University Hospital immediately after collection and were then centrifuged (lithium heparin: 4302 x *g*, 7 min, 20 °C; serum: 2500 x *g*, 10 min, 20 °C).

The fecal samples were transported to the study center at the day of the blood sampling. Each specimen was weighed, frozen and stored at -20 °C. At the end of the study, feces samples were homogenized, portioned, and the pH-value was determined. The complete 24 h-urine was transported to the study center at the day of the blood sampling. The urine volume from every participant was measured, and aliquots were frozen at -20 °C until analysis.

### Food analysis

The intakes of energy, fat, protein, carbohydrate, P, Ca, Mg and Fe from the dietary records were verified using the Prodi® 5.9 software (Nutri-Science GmbH, Freiburg, Germany).

### Blood analysis

Concentrations of plasma Ca, phosphate, Mg, Fe, ferritin, transferrin, creatinine and PTH as well as serum osteocalcin, bone specific alkaline phosphatase (BAP), 1α,25-dihydroxycholecalciferol (1,25(OH)_2_D) and 25(OH)D were ascertained according to certified methods of the Institute of Clinical Chemistry and Laboratory Medicine, Jena University Hospital.

Serum cross-linked C-terminal telopeptide of type I collagen (CTX) and N-terminal propeptide of type I procollagen (P1NP) were analyzed by electrochemilumineszenz immunoassay (Roche, Mannheim, Germany) according to manufacturer’s instruction. The intra- und inter-assay CV for CTX were <5 and <10 %, respectively (data from manual). The P1NP assay had crossreactivities <1 % to CTX, osteocalcin, PTH and 25(OH)D (data from manual). The intra- and inter-assay CV for P1NP were <5 % (data from manual). FGF23 was determined by enzyme-linked immunoabsorbant assay (Immutopics, San Clemente, USA) according to the instructions in the manual. The intra- and inter-assay CV for FGF23 assay were <5 % (data from manual).

### Feces and urine analyses

Mineral concentrations in feces were analyzed via ICP-OES as described previously [14].

The concentrations of urinary Ca, phosphate, Mg and creatinine were measured according to certified methods of the Institute of Clinical Chemistry and Laboratory Medicine, Jena University Hospital.

The concentration of desoxypyridinoline (DPD) was determined using an enzyme immune assay according to manufacturer’s instructions (Quidel Corporation, San Diego, USA). The intra- and inter-assay CV for DPD were <10 and <5 %, respectively (data from manual).

### Statistics

The primary outcomes were fasting plasma phosphate and FGF23 concentrations after  eight weeks of intervention of the whole study population (influence of gender was not determined). Sample size determination was based on a significant change in plasma phosphate concentration from 1.11 ± 0.13 (baseline) to 1.25 ± 0.15 (phosphate/calcium enriched) reported by Vervloet et al. [15]. Therefore, a sample size of 16 subjects per group achieved 95 % power to detect such change. Considering a drop-out rate and the analysis of other phosphorus metabolism related parameters sample size was set to 22 subjects per group (66 subjects for whole study).

Data analysis was performed using the statistical software package IBM SPSS Statistics 21 (SPSS Inc. IBM Company, Chicago, USA). Variance homogeneity was tested using the Levene test. The effect of time in each intervention group was tested using general linear model with repeated measurements (with pairwise comparisons by Bonferroni adjustment). The effect of supplementation between groups was tested using univariate analysis of variance followed by Bonferroni *post hoc* test. Parameters in feces were tested with paired and with unpaired Students *t*-Test within each intervention group and between intervention groups, respectively. Differences were considered significant at *p* ≤ 0.05.

All values in the text and tables are means with standard deviations.

## Results

The baseline characteristics of participants of the three intervention groups are presented in Table 1. There were no significant differences in age, BMI, 25(OH)D and CKD-EPI between intervention groups.

**Table 1:** Baseline characteristics of participants who completed the study

Parameter	P100/Ca0	P1000/Ca500	P1000/Ca1000
*n* all	20	20	22
*n* men	9	10	11
*n* women	11	10	11
Age [years]	29±8	29±6	28±6
BMI [kg/m^2^]	23±4	23±2	24±4
CKD-EPI [ml/min/173m^2^]	99±16	103±15	105±13
25(OH)D [nmol/l]	51±19	50±17	51±31

*n* = 62; data are expressed as mean ± standard deviation; CKD-EPI: Chronic Kidney Disease Epidemiology Collaboration equation for estimating the glomerular filtration rate; P1000/Ca0: 1000 mg phosphorus/0 mg calcium; P1000/Ca500: 1000 mg phosphorus/500 mg calcium; P1000/Ca1000: 1000 mg phosphorus/1000 mg calcium; 25(OH)D: 25-hydroxycholecalciferol

### Nutrient intake

There were no differences in the intakes of energy, fat, protein, and carbohydrates between study periods (placebo, four and eight weeks) and between intervention groups. The mean intakes of energy, fat, protein, and carbohydrates were 9.6 ± 2.4 MJ/d, 86.2 ± 28.5 g/d, 79.6 ± 25.5 g/d, and 249.0 ± 79.5 g/d, respectively (data not shown). Due to mineral supplementations, the intakes of Ca and P increased significantly in the respective intervention groups (Table 2). The intakes of Mg and Fe did not change between the three study periods and between the interventions. The mean Mg and Fe intakes were 322.0 ± 100.1 and 12.1 ± 3.8 mg/d, respectively (data not shown).

**Table 2:** Mean calculated intake of energy, protein, fat, carbohydrates, calcium and phosphorus during the study periods

Parameter	P1000/Ca0	P1000/Ca500	P1000/Ca1000
Energy [kJ/d]			
Placebo	8677±2329	9668±2319	9338±2791
4 weeks	9350±3125	9993±2791	9005±2270
8 weeks	8951±2388	9366±1872	9716±2598
Protein [g/d]			
Placebo	76±28	76±17	78±35
4 weeks	78±22	81±25	73±30
8 weeks	80±24	78±15	85±36
Fat [g/d]			
Placebo	76±22	92±28	84±33
4 weeks	84±33	88±28	80±31
8 weeks	79±25	79±21	91±33
Carbohydrates [g/d]			
Placebo	223±73	250±82	243±68
4 weeks	233±101	278±101	234±69
8 weeks	233±85	256±86	245±73
Calcium [mg/d]			
Placebo	887±309	893^a^±367	946^a^ ±428
4 weeks	884±299^A^	1382^b^±378^B^	1889^b^±393^C^
8 weeks	927±317^A^	1383^b^±273^B^	2008^b^ ±379^C^
Phoshorus [mg/d]			
Placebo	1232^a^±395	1312^a^±381	1306^a^±580
4 weeks	2294^b^±367	2324^b^±445	2214^b^±523
8 weeks	2307^b^±308	2276^b^±230	2401^b^±445

*n* = 62; data are expressed as mean ± standard deviation; P1000/Ca0: 1000 mg phosphorus/0 mg calcium; P1000/Ca500: 1000 mg phosphorus/500 mg calcium; P1000/Ca1000: 1000 mg phosphorus/1000 mg calcium; ^a^ ^b^ mean values within a column with dissimilar superscript lower case letters are significantly different (*p* ≤ 0.05); ^A^ ^B^ mean values within a row with dissimilar superscript capital letters are significantly different (*p* ≤ 0.05); effect of time was tested using general linear model with repeated measurements (with pairwise comparisons based on Bonferroni); effect of supplementation was tested using univariate analysis of variance followed by Bonferroni *post hoc* test

### Minerals

**Fig. 3:**
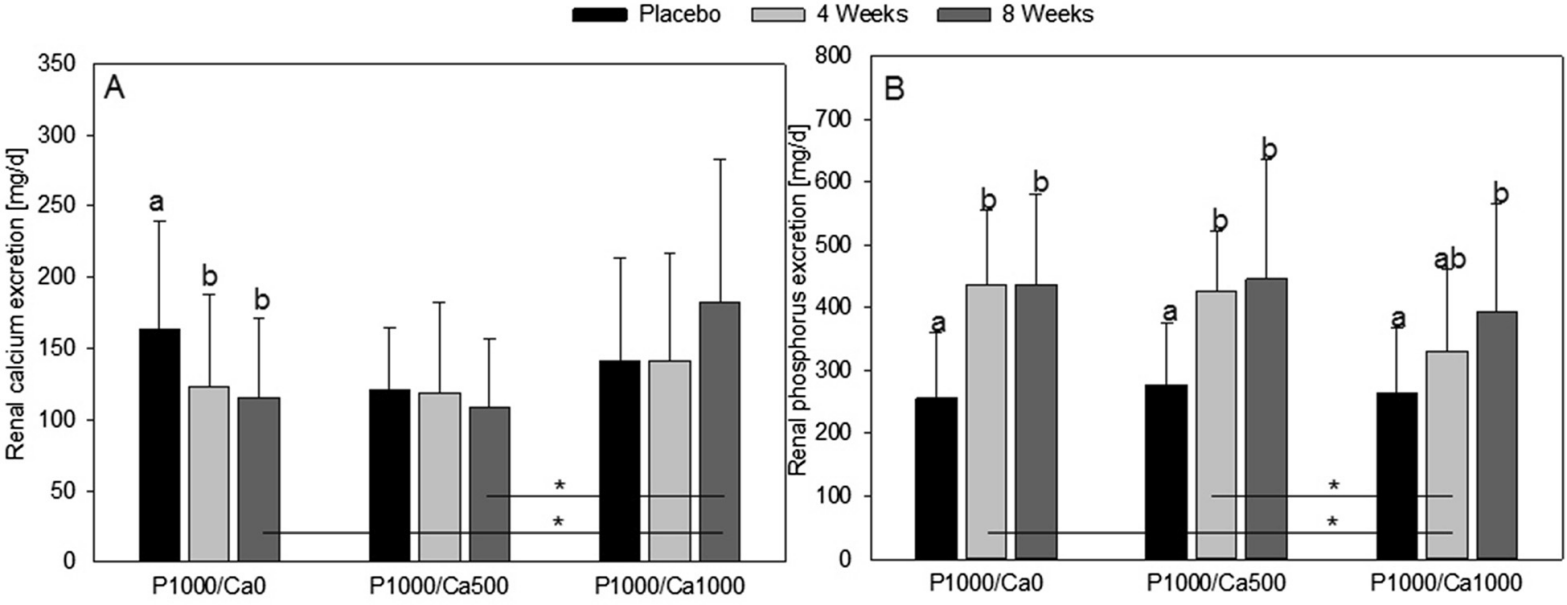
Renal calcium (**a**) and phosphorus (**b**) excretion after nutritional intervention with phosphorus and calcium. **a** *n* = 59; **b** *n* = 62; data are expressed as mean + standard deviation; mean values within an intervention group with dissimilar superscript letters are significantly different (*p* ≤ 0.05); mean values without superscripts have no significant differences; * significant different (*p* ≤ 0.05); effect of time was tested using general linear model with repeated measurements (with pairwise comparisons based on Bonferroni); effect of supplementation was tested using univariate analysis of variance followed by Bonferroni *post hoc* test; P1000/Ca0: 1000 mg phosphorus/0 mg calcium; P1000/Ca500: 1000 mg phosphorus/500 mg calcium; P1000/Ca1000: 1000 mg phosphorus/1000 mg calcium

After eight weeks of P1000/Ca500 intervention the plasma Ca concentration was significant higher compared to four weeks of intervention (Table 3; *p* = 0.047). The renal Ca excretion significantly decreased after four (*p* = 0.001) and eight (*p* = 0.029) weeks of P1000/Ca0 intervention compared to placebo (Fig. 3). Fecal Ca concentrations were significantly increased after eight week intervention compared to placebo in the P1000/Ca500 (*p* ≤ 0.001) and P1000/Ca1000 (*p* ≤ 0.001) groups (Table 3).

**Table 3:** Mean plasma and feces concentrations and renal excretion of minerals during the study periods

Parameter	P1000/Ca0	P1000/Ca500	P1000/Ca1000
Plasma calcium [mmol/l]			
Placebo	2.36±0.08	2.36^ab^±0.06	2.36±0.08
4 weeks	2.34±0.07	2.35^a^±0.05	2.38±0.08
8 weeks	2.37±0.09	2.39^b^±0.07	2.39±0.09
Fecal calcium [mg/g feces]			
Placebo	9±5	7±3*	8±3*
8 weeks	8±4	11±5	12±6
Plasma phosphate [mmol/l]			
Placebo	1.20±0.23	1.28±0.16	1.19±0.23
4 weeks	1.19±0.19	1.24±0.14	1.23±0.20
8 weeks	1.18±0.23	1.20±0.12	1.23±0.19
Fecal phosphorus [mg/g feces]			
Placebo	5±2*	5±2*	6±3*
8 weeks	8±4	8±4	8±3
Plasma magnesium [mmol/l]			
Placebo	0.82±0.06	0.83±0.04	0.82±0.05
4 weeks	0.82±0.05	0.83±0.04	0.82±0.05
8 weeks	0.83±0.05	0.84±0.05	0.81±0.05
Urine magnesium [mg/d]			
Placebo	113^a^±41	110±30	106±48
4 weeks	89^b^±39	103±36	91±42
8 weeks	84^b^±32	103±53	106±56
Fecal magnesium [mg/g feces]			
Placebo	2±1	2±1	2±1
8 weeks	2±1	2±1	2±1

*n* = 62 for plasma and minerals in urine; *n* = 61 for fecal minerals; data are expressed as mean ± standard deviation; P1000/Ca0: 1000 mg phosphorus/0 mg calcium; P1000/Ca500: 1000 mg phosphorus/500 mg calcium; P1000/Ca1000: 1000 mg phosphorus/1000 mg calcium; ^a b^ mean values within a column with dissimilar superscript lower case letters are significantly different (*p* ≤ 0.05); * significant different to eight weeks within each group (*p* ≤ 0.05); effect of time was tested using general linear model with repeated measurements (with pairwise comparisons based on Bonferroni); fecal minerals were tested with paired and with unpaired Students *t*-Test within each intervention group and between intervention groups, respectively.

Plasma phosphate concentrations did not change significantly due to any of the interventions. After all interventions renal phosphate concentrations significantly increased after eight weeks compared to placebo (P1000/Ca0 *p* ≤ 0.001, P1000/Ca500 *p* = 0.007, P1000/Ca1000 *p* = 0.008) (Fig. 3). In the P1000/Ca0 and P1000/Ca500 groups renal phosphate excretions increased after four weeks as well (both *p* ≤ 0.001) (Fig. 3). Fecal P concentrations significantly increased after eight weeks of all interventions compared to placebo (P1000/Ca0 ≤ 0.001, P1000/Ca500 ≤ 0.001, P1000/Ca1000 *p* = 0.012) (Table 3).

Plasma Mg concentrations did not change due to any intervention; whereas renal Mg excretions decreased significantly after four and eight weeks of P1000/Ca0 intervention (Table 3; four weeks *p* ≤ 0.027, eight weeks *p* ≤ 0.001).

None of the interventions changed plasma Fe and ferritin concentrations as well as transferrin saturations (Table 4). Plasma transferrin concentrations increased significantly after eight weeks of intervention with P1000/Ca500 compared to placebo and four weeks (Table 4; placebo *p* ≤ 0.005, four weeks *p* ≤ 0.037).

**Table 4:** Parameters of iron metabolism during the study periods

Parameter	P1000/Ca0	P1000/Ca500	P1000/Ca1000
Plasma iron [pmol/l]			
Placebo	17.1±6.5	16.9±7.4	17.1±8.9
4 weeks	15.3±5.8	16.0±7.0	17.4±8.0
8 weeks	16.2±6.2	16.2±5.6	17.0±7.5
Plasma transferrin [g/l]			
Placebo	2.7±0.5	2.9^a^±0.6	2.9±0.6
4 weeks	2.8±0.4	2.9^a^±0.5	3.0±0.6
8 weeks	2.8±0.6	3.1^b^±0.7	3.0±0.7
Transferrin saturation [%]			
Placebo	22.8±13.3	25.2±13.1	26.6±11.4
4 weeks	22.2±8.6	22.5±9.7	24.6±12.5
8 weeks	24.0±10.6	21.7±7.3	23.9±12.9
Plasma ferritin [pg/d]			
Placebo	61.8±73.9	62.9±76.7	57.9±51.7
4 weeks	59.2±61.4	58.2±70.6	59.0±49.8
8 weeks	68.6±66.7	63.0±79.4	52.0±41.1
Fecal iron [pg/g feces]			
Placebo	90±40	70±30	80±20
8 weeks	70±30	70±50	60±30

*n* = 62 for plasma parameter; *n* = 61 for fecal iron; data are expressed as mean ± standard deviation; P1000/Ca0: 1000 mg phosphorus/0 mg calcium; P1000/Ca500: 1000 mg phosphorus/500 mg calcium; P1000/Ca1000: 1000 mg phosphorus/1000 mg calcium; ^a b^ mean values within a column with dissimilar superscript lower case letters are significantly different (*p* ≤ 0.05); effect of time was tested using general linear model with repeated measurements (with pairwise comparisons based on Bonferroni); fecal iron was tested with paired and with unpaired Students *t*-Test within each intervention group and between intervention groups, respectively

### Phosphate-metabolism related hormones

After eight weeks of P1000/Ca0 supplementation 1,25(OH)_2_D concentration in plasma increased significantly compared to placebo (*p* = 0.047) (Table 5).

**Table 5:** Mean concentrations of plasma and serum hormones and bone metabolism markers during the study periods

Parameter	P1000/Ca0	P1000/Ca500	P1000/Ca1000
Serum 1,25(OH)2D [pmol/l]			
Placebo	123.6^a^ +31.9	118.2±37.3	118.1±30.9
4 weeks	126.7^ab^±32.1	132.5±45.3	122.3±39.3
8 weeks	143.8^b^±31.5	138.0±47.2	123.6±44.4
Serum 25(OH)D [nmol/l]			
Placebo	59.3^a^±20.7	60.8^a^±20.0	64.0^ab^+/-36.8
4 weeks	66.7^a^±18.3	64.9^a^±16.4	62.7^a^±24.7
8 weeks	83.1^b^±19.6	82.7^b^±22.5	81.0^b^±31.1
Plasma PTH [ng/l]			
Placebo	20.0±8.1	26.2±11.8	22.2±7.0
4 weeks	20.7±6.6	24.1±7.3	21.1±6.4
8 weeks	21.0±6.7	25.7±9.5	19.9±8.2
Serum osteocalcin [ng/ml]			
Placebo	27.6±9.1	29.7^a^±11.3	26.6^a^±8.6
4 weeks	25.8±9.0	27.8^ab^±12.1	23.4^ab^±7.8
8 weeks	25.3±9.2	25.0^b^±11.7	22.3^b^±9.0
Serum BAP [ng/ml]			
Placebo	11.1±4.3	10.4^a^±2.9	11.4^a^±6.8
4 weeks	10.7±4.3	9.1^b^±2.5	10.1^b^±5.7
8 weeks	10.6±4.4	9.6^ab^±2.8	9.6^b^±5.4
Plasma CTX [ng/ml]			
Placebo	0.58±0.25	0.54^a^±0.18	0.52^a^±0.21
4 weeks	0.63±0.33	0 49^ab^±0.23	0.42^b^±0.20
8 weeks	0.60±0.31	0.44^b^±0.20	0.43^b^±0.22
Plasma P1NP [ng/ml]			
Placebo	64.77±32.31	61 13±31.47	54.44±16.68
4 weeks	65.16±30.0	63.58±32.48	56.85±24.09
8 weeks	65.61±29.69	57.67±32.25	52.33±19.26
Urine DPD [pmol/mol Creatinine]			
Placebo	3.9±1.7	4.4±2.6	4.3±1.7
4 weeks	4.1±1.7	3.9±1.1	3.9±1.5
8 weeks	3.9±1.4	3.7±1.0	3.9±1.4

*n* = 58 for plasma P1NP and CTX; *n* = 60 for 25(OH)D; *n* = 61 for plasma osteocalcin. BAP. PTH and serum 1.25(OH)_2_D; *n* = 62 for urine DPD; data are expressed as mean ± standard deviation; BAP: bone specific alkaline phosphatase; CTX: cross-linked C-terminal telopeptide of type I collagen; DPD: desoxypyridinoline; PTH: parathyroid hormone; P1NP: N-terminal propeptide of type I procollagen; P1000/Ca0: 1000 mg phosphorus/0 mg calcium; P1000/Ca500: 1000 mg phosphorus/500 mg calcium; P1000/Ca1000: 1000 mg phosphorus/1000 mg calcium; 25(OH)D: 25-hydroxycholecalciferol; 1.25(OH)_2_D: 1α,25-dihydroxycholecalciferol; ^a b^ mean values within a column with dissimilar superscript lower case letters are significantly different (*p* ≤ 0.05); effect of time was tested using general linear model with repeated measurements (with pairwise comparisons based on Bonferroni)

In the P1000/Ca0 and P1000/Ca500 supplemented groups 25(OH)D plasma concentrations increased significantly after eight weeks of intervention compared to placebo and four weeks (all *p* ≤ 0.001). The 25(OH)D plasma concentration was significantly higher after eight weeks P1000/Ca1000 intervention compared to four weeks P1000/Ca1000 intervention (*p* = 0.004) (Table 5). This is mainly caused by the study time between March and July, in which the endogenous Vitamin D production via UV-light increased. Similar results were shown in the placebo group of another human intervention study [16].

The concentrations of PTH in plasma did not change due to any of the three interventions (Table 5).

**Fig. 4:**
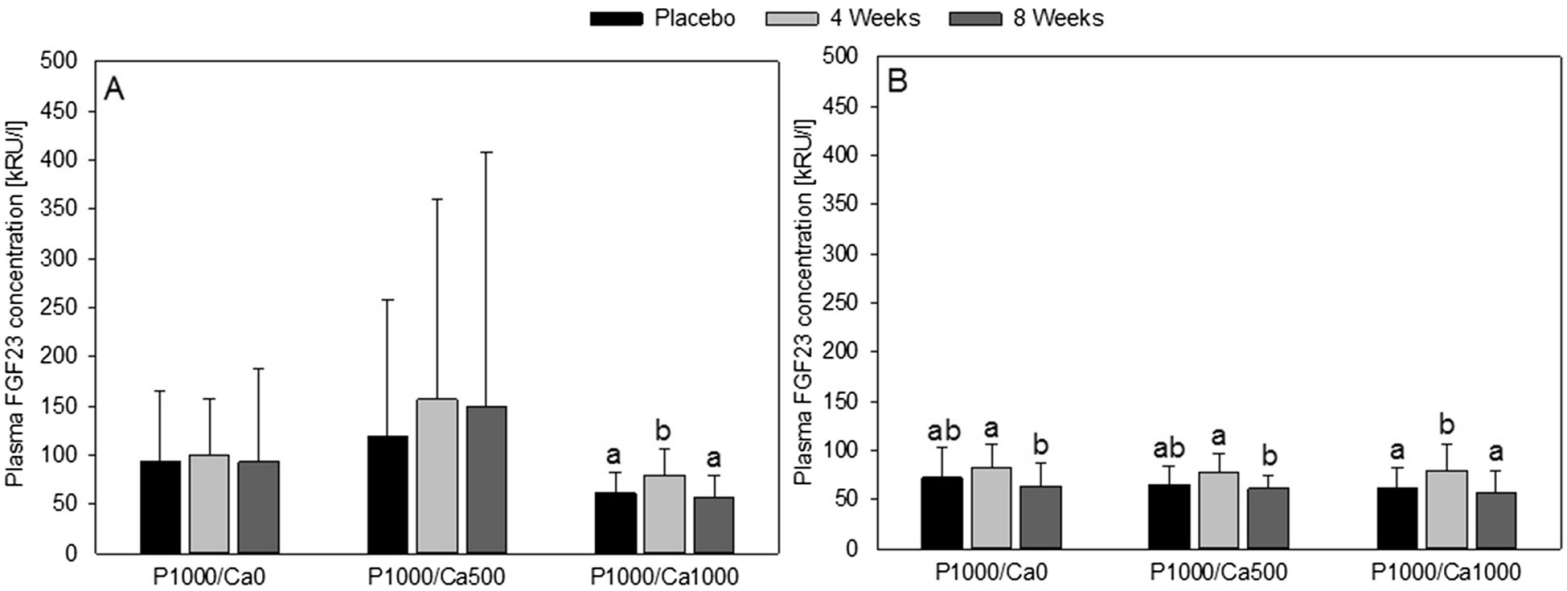
Plasma concentrations of fibroblast growth factor 23 after nutritional intervention with phosphorus and calcium. **a** *n* = 61 all subjects; (**b**) *n* = 56 subjects with concentrations above 300 kRU/l in at least one study period; data are expressed as mean + standard deviation; FGF23: fibroblast growth factor 23; mean values within an intervention group with dissimilar superscript letters are significantly different (*p* ≤ 0.05); mean values without superscripts have no significant differences; effect of time was tested using general linear model with repeated measurements (with pairwise comparisons based on Bonferroni); effect of supplementation was tested using univariate analysis of variance followed by Bonferroni *post hoc* test; P1000/Ca0: 1000 mg phosphorus/0 mg calcium; P1000/Ca500: 1000 mg phosphorus/500 mg calcium; P1000/Ca1000: 1000 mg phosphorus/1000 mg calcium

Regarding the FGF23 concentrations, the standard deviations in the P1000/Ca0 und P1000/Ca500 interventions groups were disproportionately high compared to P1000/Ca1000 (Fig. 4). This was due to five subjects with very high FGF23 concentrations. Therefore, we excluded all subjects with concentrations above 300 kRU/l in at least one study period (two subjects P1000/Ca0, three subjects P1000/Ca500). After the exclusion the standard deviations were comparable between all three intervention groups. After all interventions the FGF23 concentrations were significantly higher after four weeks compared to eight weeks of intervention (P1000/Ca0 *p* = 0.023, P1000/Ca500 *p* = 0.005, P1000/Ca1000 *p* = 0.001). In the P1000/Ca1000 group the concentration of FGF23 was significantly higher after four weeks compared to placebo, too (*p* = 0.020).

### Bone metabolism markers

Due to supplementation of P and Ca (P1000/Ca1000, P1000/Ca500) the concentrations of the bone formation marker osteocalcin significantly decreased after eight weeks of intervention compared to placebo (P1000/Ca500 *p* = 0.008; P1000/Ca1000 *p* ≤ 0.001; Table 5). Compared to placebo BAP concentration significantly decreased in the P1000/Ca500 supplemented group after four weeks of intervention (*p* ≤ 0.007). In the P1000/Ca1000 supplemented group, the BAP concentration significantly decreased after four and eight weeks of intervention compared to placebo (four weeks *p* = 0.005; eight weeks *p* = 0.003).

The concentrations of CTX, a marker of bone resorption, decreased significantly after four and eight weeks after P1000/Ca1000 intervention (four weeks *p* ≤ 0.005, eight weeks *p* = 0.05). After P1000/Ca500 intervention the CTX concentration significantly decreased after eight weeks of intervention compared to placebo (*p* = 0.003). Plasma P1NP and urine DPD concentrations did not change due to any of the three interventions.

## Discussion

The epidemiological studies from Tonelli et al. [3] and Dhingra et al. [2] only determined an association between serum phosphate concentrations and the risk for cardiovascular diseases, but not the influence of the dietary P intake. Chang et al. [17] showed that high P intakes (>1400 g/d), but not fasting serum phosphorus concentrations are associated with an increased mortality in a healthy US population. The authors concede that additional studies are necessary to determine whether this relation is causal.

The results of the present study show clearly that additional intake of 1000 mg P/d did not influence fasting plasma phosphate concentrations of healthy adults after eight weeks of intervention, independent of Ca intake. This is in accordance with other studies, which examined among other things fasting phosphate concentrations [11, 18–20]. By looking at short-term interventions, it should be noticed that serum phosphate concentrations increased after high P intake during the day, but returned to fasting concentrations [20–24]. The phosphate homeostasis in healthy subjects comprises obviously a regulation of acute P loads. Since renal P excretions increased after additional P intakes in the present study, it can be hypothesized that elimination of P via the kidney occurred. Interestingly, the fecal P concentrations increased as well, which indicates the formation of amorphous calcium phosphate in the intestine [16, 25, 26]. Noteworthy, the renal excretion of Ca decreased after P1000/Ca0 intervention, while the renal Ca excretions remained stable in the Ca-supplemented intervention groups. This indicates a negative influence of a high P and a normal Ca diet on Ca balance and is in accordance with Kemi et al. [27], who reported a disturbed Ca metabolism by a Ca:P ratio below 0.5 (present study P1000/Ca0 0.4). However, the other two intervention groups with Ca and P (P1000/Ca500 and P1000/Ca1000) showed no negative effect on Ca balance since plasma Ca concentrations increased and renal excretions remained unchanged. Our results and the existing literature give evidence for maintenance of a positive Ca balance, a high P intake has to be accompanied by a high Ca intake [27–29].

FGF23 is a major factor in the regulation of phosphate homeostasis, especially the regulation of blood phosphate after dietary intake [5]. Interestingly, the plasma FGF23 is discussed as a risk factor for mortality in patients with CKD [30]. Gutiérrez et al. [30] concluded that increased FGF23 could be a new biomarker for death in early CKD, when phosphate concentration is still normal. Mortality-associated FGF23 concentrations are far away the FGF23 concentration of healthy subjects in the present study. This is not surprising since patients with CKD have 3-fold higher FGF23 concentrations compared to healthy subjects [13]. In our study were five subjects with FGF23 concentrations above 300 kRU/l in at least one study period. These subjects showed regular P and Ca parameters and did not suffer from CKD. The results of the remaining 56 subjects were similar in the three intervention groups. The FGF23 concentration increased on average by 14 kRU/l (17 %) after four weeks of supplementation compared to placebo (only significant for P1000/Ca1000) and decreased by 19 kRU/L (33 %) after eight weeks compared to four weeks of supplementation. Burnett et al. [31] showed an increase of approximately 37 % for FGF23 (extracted from Fig. 2, taking the reported baseline value into account: mean increase of 21 kRU/l) within five days of P supplementation. Similar, but not significant, results found Ferrari et al. [20]. It seems that FGF23 temporarily increases after P load, but decreases in adaption to the diet. To the best of our knowledge, there is no study which determined FGF23 after intervention longer than four weeks. We could show for the first time that an increase of FGF23 is not a permanent effect. Therefore, we assume that other mechanism keeps phosphate concentration in homeostasis. Since PTH and 1,25(OH)_2_D are the best known phosphate regulators it is necessary to focus these two. In the present study, PTH did not change after the three interventions. 1,25(OH)_2_D increased in all intervention groups after eight weeks of supplementation compared to placebo, but significantly only in P1000/Ca0. This is little surprising since literature reports that high P intake results in PTH and FGF23 increases, which cause phosphaturia [29]. PTH stimulates and FGF23 inhibits 1,25(OH)_2_D synthesis, resulting in an increase and decrease of P absorption, respectively. Additionally, FGF23 in turn inhibits PTH [29]. The unchanged fasting PTH concentrations after high P intake are in accordance with Grimm et al. [19] and Ix et al. [13], but not with other human intervention studies, which showed an increase in fasting PTH concentrations [18, 32–34]. The main reason for the differences between the above-mentioned studies and the present one is probably the different intervention time (present study: 8 weeks; other studies: maximum 28 days).

The determined bone formation (osteocalcin and BAP) and resorption markers (CTX) decreased significantly in the Ca-supplemented groups. The P supplementation alone did not change the markers significantly. According to Coates et al. [35] the day-to-day variation should be taken into account for interpretation of bone markers, thus a significant change is generally determined as 2.8 times the biological variation. Due to the fact that the measured bone markers generally vary between 8–12 %, the change should be greater than approximately 30 % in order to get clinical relevance [35]. However, the determined markers of bone metabolism in the present study changed only approximately 20 %. Therefore, we could not observe a clinically relevant effect on bone remodeling after a high P intake in healthy subjects.

After P supplementation but without Ca supplementation, renal Mg excretion decreased. This outcome indicates a decreased absorption of Mg in the gut due to high P intake and is in accordance with Brink et al. [36]. These authors showed in an rat experiment that Mg forms insoluble complexes with Ca and phosphate. Greger et al. [37] showed that significantly less Mg was excreted via urine of men following high P consumption (2443 mg P) compared to moderate P diets (843 mg P). But the authors concluded, that the apparent Mg retention and thus the utilization of Mg was not affected. Transferrin concentration in plasma slightly increased due to  eight weeks of P1000/Ca500 intervention compared to placebo. The other iron metabolism marker did not change due to the interventions. An interaction of a calcium intervention with iron was shown in mostly short-term studies [38–40]. In a long-term study of Minihane and Fairweather-Tait [41] no effect of iron metabolism after calcium supplementation was shown. The influence of phosphate intake on iron metabolism was not yet investigated. Thus, it should be taken into consideration, that a high P intake may unbalance Mg and iron metabolism.

Throughout the eight weeks of P supplementation, 16 % of subjects reported intestinal disturbances (diarrhea, flatulence, stomachache and/or constipation). This is a consequence of an osmotic effect in the intestine due to the high P concentration [19].

A potential limitation of the study is the determination of fasting blood parameters instead of postprandial ones, because it is known that phosphate concentrations change differently throughout the day, depending on the phosphors intake [22]. However, the above-mentioned epidemiological studies [2, 3, 17] used fasting measurements and the present study could show that eight weeks of high phosphorus intake could not change fasting phosphate concentration. Therefore, it should be taken into consideration that the higher risk for mortality and cardiovascular disease does not comes solely from the diet. It is most likely an effect of interaction between genetics, other not recognized diseases (e.g. kidney) and the diet.

Another limitation factor could be the potential declining compliance of the subjects after ten weeks of study. Nevertheless, due to the increased renal excretion as well as fecal concentration of P after eight weeks of P intervention the authors conclude a persistent compliance.

## Conclusions

In all, our study showed that a high P intake did not influence fasting phosphate plasma concentrations in healthy adults. A high P intake without adequate Ca supplementation seems to have a negative impact on Ca metabolism. Thus, it can be stated that a well-balanced Ca:P ratio is an important prerequisite for a normal metabolism of Ca. There is only a temporary increase of FGF23 after P supplementation. Within eight weeks of a high P intervention, plasma concentrations of FGF23 remained within the normal range.

## Abbreviations

P: Phosphorus
Ca: Calcium
CTX: Cross-linked C-terminal telopeptide of type I collagen
CKD: Chronic kidney disease
BAP: Bone specific alkaline phosphatase
DPD: Desoxypyridinoline
PTH: Parathyroid hormone
P1NP: n-terminal propeptide of type I procollagen
1,25(OH)2D: 1α,25-dihydroxycholecalciferol
25(OH)D: 25-hydroxycholecalciferol
FGF23: Fibroblast growth factor 23
CKD-EPI: Chronic Kidney Disease Epidemiology Collaboration equation for estimating the glomerular filtration rate
Mg: Magnesium
Fe: Iron
